# Single Versus Double Dose Praziquantel Comparison on Efficacy and *Schistosoma mansoni* Re-Infection in Preschool-Age Children in Uganda: A Randomized Controlled Trial

**DOI:** 10.1371/journal.pntd.0003796

**Published:** 2015-05-26

**Authors:** Allen Nalugwa, Fred Nuwaha, Edridah Muheki Tukahebwa, Annette Olsen

**Affiliations:** 1 Child Health and Development Centre, Makerere University, Kampala, Uganda; 2 Disease Control and Prevention, Makerere University, Kampala, Uganda; 3 Vector Control Division, Ministry of Health, Kampala, Uganda; 4 Department of Veterinary Disease Biology, University of Copenhagen, Frederiksberg, Denmark; Swiss Tropical and Public Health Institute, SWITZERLAND

## Abstract

**Background:**

*Schistosoma mansoni* infection is proven to be a major health problem of preschool-age children in sub-Saharan Africa, yet this age category is not part of the schistosomiasis control program. The objective of this study was to compare the impact of single and double dose praziquantel (PZQ) treatment on cure rates (CRs), egg reduction rates (ERRs) and re-infection rates 8 months later, in children aged 1-5 years living along Lake Victoria, Uganda.

**Methodology/Principal Findings:**

Infected children (n= 1017) were randomized to receive either a single or double dose of PZQ. Initially all children were treated with a single standard oral dose 40 mg/kg body weight of PZQ. Two weeks later a second dose was administered to children in the double dose treatment arm. Side effects were monitored at 30 minutes to 24 hours after each treatment. Efficacy in terms of CRs and ERRs for the two treatments was assessed and compared 1 month after the second treatment. Re-infection with *S*. *mansoni* was assessed in the same children 8 months following the second treatment. CRs were non-significantly higher in children treated with two 40 mg/kg PZQ doses (85.5%; 290/339) compared to a single dose (83.2%; 297/357). ERRs were significantly higher in the double dose with 99.3 (95%CI: 99.2-99.5) compared with 98.9 (95%CI: 98.7-99.1) using a single dose, (P = 0.01). Side effects occurred more frequently during the first round of drug administration and were mild and short-lived; these included vomiting, abdominal pain and bloody diarrhea. Overall re-infection rate 8 months post treatment was 44.5%.

**Conclusions:**

PZQ is efficacious and relatively safe to use in preschool-age children but there is still an unmet need to improve its formulation to suit small children. Two PZQ doses lead to significant reduction in egg excretion compared to a single dose. Re-infection rates with *S*. *mansoni* 8 months post treatment is the same among children irrespective of the treatment regimen.

## Author Summary

Intestinal schistosomiasis is a parasitic water borne neglected tropical disease of considerable public health relevance in the tropics and subtropics. In Uganda, approximately 20 million people are at risk of being infected with *S*. *mansoni*, which causes intestinal schistosomiasis, and 4 million individuals are estimated to be infected. Currently, schistosomiasis control using PZQ chemotherapy is mainly focusing on school-age children (age 6–19 years) and to those adults considered to have the highest risk of infection. So far preschool-age children are not targeted in schistosomiasis preventive chemotherapy campaigns due to the limited documentation on the safety of PZQ in this age group. However, a number of studies have revealed that children less than 6 years of age get infected with *S*. *mansoni* usually through bathing, playing or swimming in schistosome-infested waters and are at higher risk of infection than previously thought. This study compared efficacy of PZQ treatment in terms of CRs and ERRs using a single and double dose among children aged 1–5 years, living along Lake Victoria in eastern Uganda. The effect of the two different regimes on *S*. *mansoni* re-infection 8 months post treatment was also assessed.

## Introduction

Schistosomiasis is a parasitic water borne neglected tropical disease [1, 2] of considerable public health relevance in the tropics and subtropics [3, 4]. It is estimated that 200 million people are infected with schistosomiasis worldwide with 85% of the disease burden found in Sub-Saharan Africa [5]. In Uganda, approximately 20 million people are at risk of being infected with intestinal schistosomiasis caused by *S*. *mansoni* and 4 million individuals are estimated to be infected [6]. Currently, the focus of schistosomiasis control by preventive chemotherapy is on school-age children of 6–19 years as this category has the highest risk of infection [7, 8]. Mass drug administration (MDA) with PZQ for schistosomiasis is the main approach adopted by the Uganda national schistosomiasis control program to reduce related morbidity in school-age children and adults. A number of studies on children under 6 years of age have, however, revealed that these children are at higher risk of schistosomiasis infection than previously thought [9–12]. Young children living on lakeshores or irrigated land actively get infected with schistosome parasites usually through bathing, playing or swimming in schistosome-infested waters. These children may also get exposed passively to infective water when bathed with lake water, which are carried back home [10].

Preschool-age children are not yet targeted in schistosomiasis preventive chemotherapy campaigns. The exclusion of preschool-age children (<6 years of age) from mass treatment programmes for control of schistosomiasis has been justified by the limited documentation on the safety of PZQ in this age group [13, 14]. Although PZQ is recommended for individuals aged 4 years and above [8], there is no suitable formulation of PZQ for young children [15]. Meanwhile, studies conducted in some schistosomiasis endemic countries (Mali, Niger, Sudan, Uganda and Zimbabwe) have documented that it is safe and efficacious to treat children (<6 years) with PZQ [16, 17]. Based on the same findings, it has been recommended that young children who live in schistosomiasis endemic areas should be considered for treatment with PZQ during childhood [16].

PZQ is a large white tablet containing 600mg of active ingredient [18] and is, despite its large size and bitter taste, the drug still being used to treat human schistosomiasis on a large scale [19–21]. The inactive distomer (*S*-PZQ) contributes to the bitter taste and doubles the size of the tablets [21]. It is the drug of choice because it is effective on all schistosome species, cheap, safe and has minimal side effects [20, 22]. The standard recommended dose is a single oral treatment of 40 mg/kg body weight [8, 23, 24] sufficient to achieve CRs of 60–90% [25, 26]. Upon oral intake, PZQ penetrates the tegument and rapidly moves through the tissues of schistosomes causing muscle contraction and damage [25, 27]. PZQ attacks only the mature schistosome worms but not the immature stages [20] and has therefore no effect on recent infections [28, 29]. Consequently it is recommended to administer a second dose of PZQ two weeks after a first dose in order to kill the worms which were immature during the first treatment and hence, increase CRs. This study was designed to compare the efficacy of PZQ in terms of CRs and ERRs using single and double dose regimens and its effect on *S*. *mansoni* re-infection 8 months post treatment in children aged 1–5 years living along Lake Victoria in eastern Uganda.

## Methods

### Study area and design

**Fig 1:**
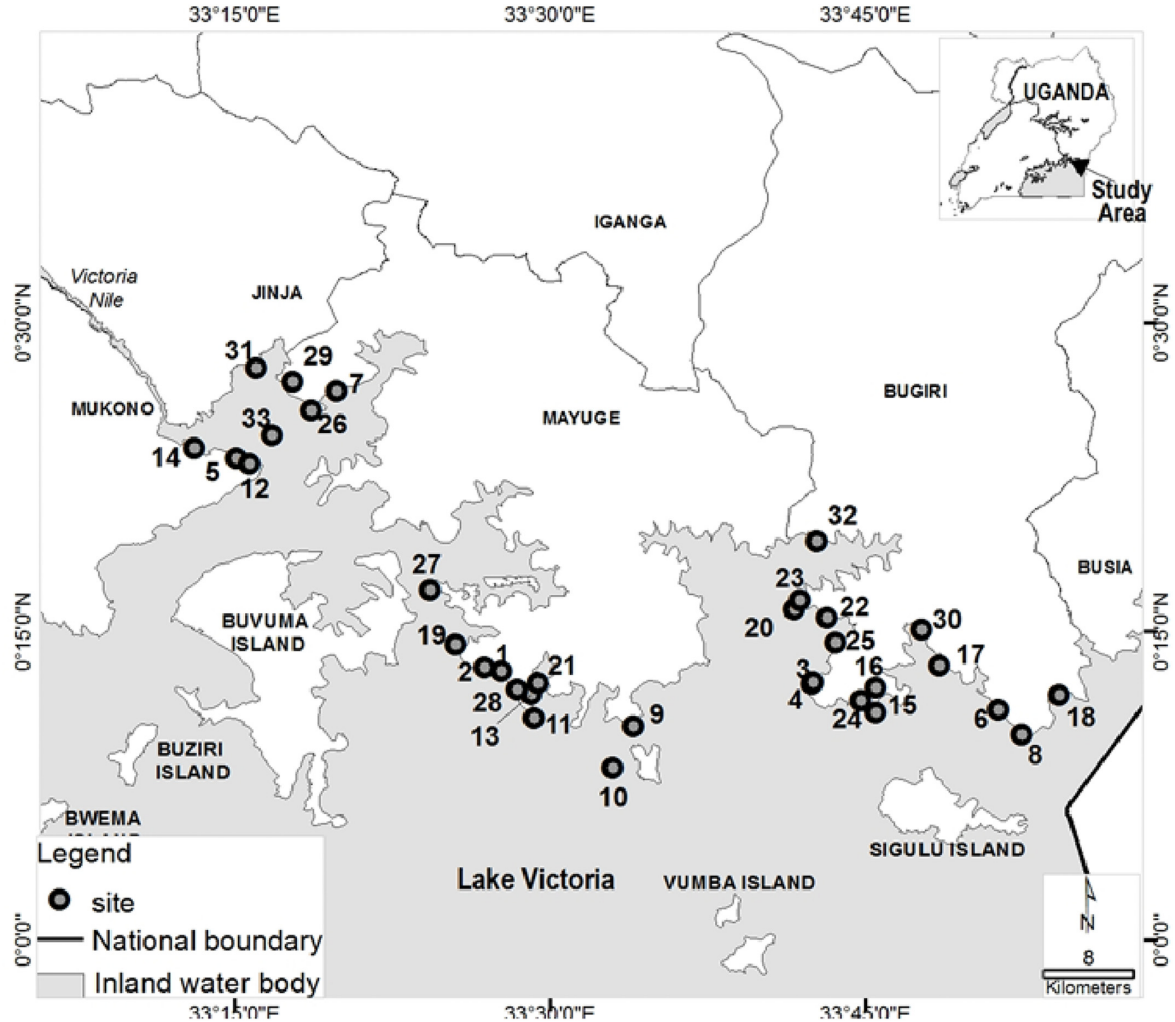
Map showing study sites along Lake Victoria, eastern Uganda. Study Sites1-Bukagabo A; 2-Bukagabo B; 3-Bumeru A; 4-Bumeru B; 5-Busana; 6-Busiro; 7-Busuyi; 8-Buyondo; 9-Bwondha; 10-Gori Island; 11-Kabuka; 12-Kalindi; 13-Kayanja; 14-Kikondo; 15-Lubango A; 16-Lubango B; 17-Lufudo; 18-Lugala; 19-Malindi; 20-Maruba; 21-Masaka; 22-Matiko; 23-Mpanga; 24-Mulwanda; 25-Musoli; 26-Musoli A; 27-Namoni; 28-Nango; 29-Ntinkalu; 30-Sidome; 31-Wairaka; 32-Wakawaka; 33-Kisiima Island.

The study was carried out in 33 sites (31 shoreline communities and 2 islands) along Lake Victoria in eastern Uganda (Fig 1); where different epidemiological studies reported high prevalence of *S*. *mansoni* infections [11, 30–33]. Malacological surveys have also confirmed that *Biomphalaria choanomphala*, one of the main freshwater snail intermediate host of *S*. *mansoni*, exists in high numbers along Lake Victoria shorelines in Uganda [34]. The major activities carried out by these communities are fishing and subsistence farming. The lake is the major source of water for domestic use and the communities are characterized by poor sanitation.

**Fig 2:**
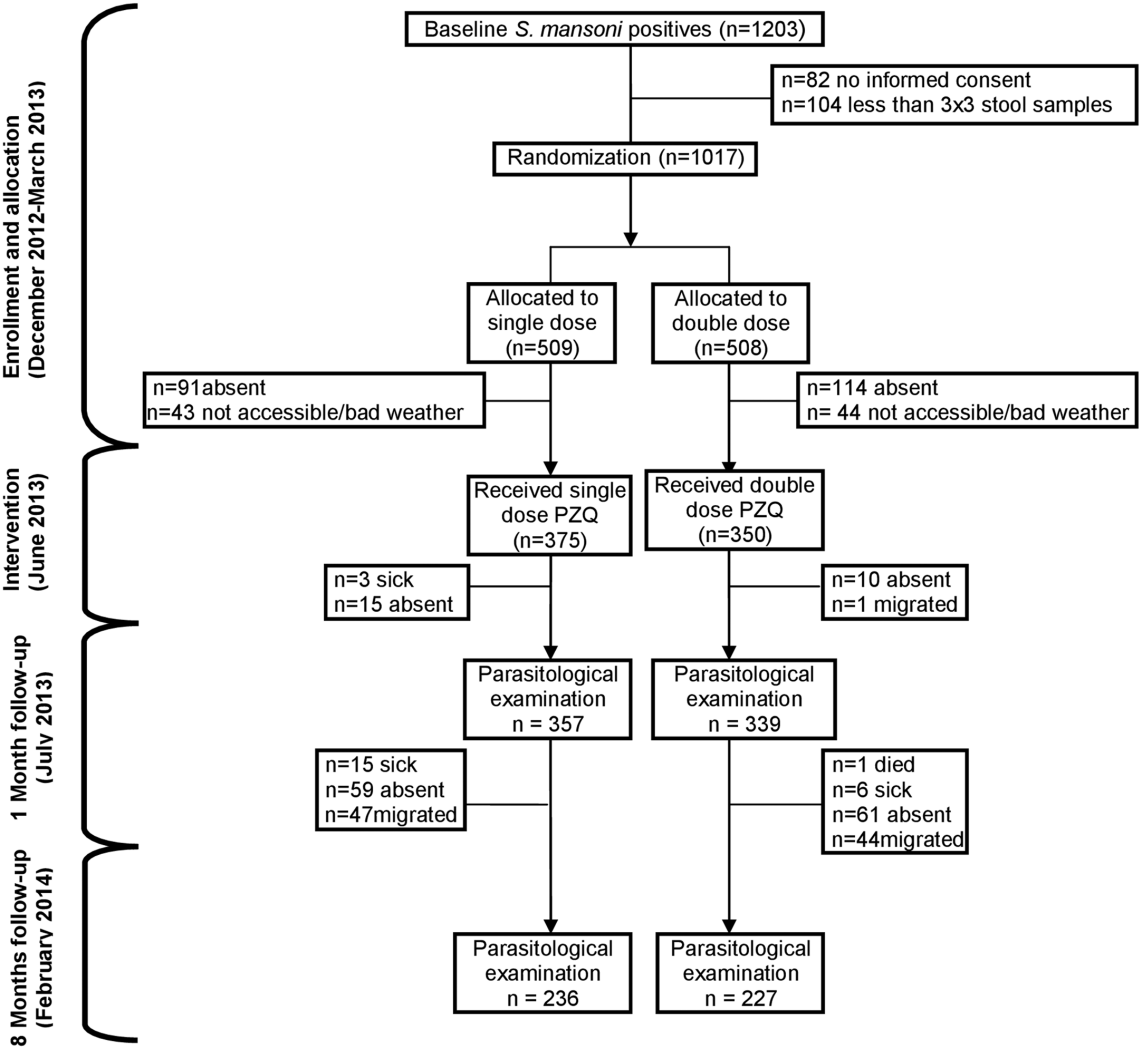
Enrollment, randomization, follow-up, and inclusion in the final analysis in the study comparing single and double dose PZQ treatment groups.

We designed a randomized clinical trial (ClinicalTrial.gov, identifier: NCT01901484) with two treatment arms (single versus spaced double dose) of PZQ with follow-up assessments at month 1 and 8 months post treatment (Fig 2). Following a baseline parasitological survey, all the children tested positive for *S*. *mansoni* eggs and who provided three stool specimens and informed consent from their parents were recruited into the study. Recruited children were then individually randomized (by an independent statistician) to single and double dose PZQ treatment groups by computer random number generation in Stata/IC 12.0. Baseline characteristics of the two treatment groups are summarized in Table 1. Age, sex, distribution of intensity levels (light, moderate and heavy) and sampled sites were comparable between the two treatment groups. Children were orally administered a single dose of PZQ (40 mg/kg of body weight) at baseline and another similar dose two weeks later to those in the double dose treatment group. Eligibility was limited to preschool-age-aged children (1–5 years). Follow-up surveys were carried out 1 month and 8 months after the second treatment with PZQ to assess efficacy and re-infection, respectively.

**Table 1:** Baseline characteristics for preschool-age children who were infected with *Schistosoma mansoni* and who were randomized to single and double dose treatments (n = 1017).

Characteristic	Single dose (n = 509) % (95% CI)	Double dose (n = 508) % (95% CI)	Statistic test	P value
**Sex**				
Girl	50.7 (46.3–55.0)	45.7 (41.3–50.0)	χ ^2^ = 2.56	0.11
Boy	49.3 (45.0–53.7)	54.3 (50.0–58.7)		
**Intensity of infection (epg)**				
Light (1–99)	59.7 (55.5–64.0)	56.3 (52.0–60.6)	χ ^2^ = 2.275	0.32
Moderate (100–399)	23.6 (19.9–27.3)	23.4 (19.7–27.1)		
Heavy (≥ 400)	16.7 (13.5–19.9)	20.3 (16.8–23.8)		
**Sampled village/site**				
Island	3.9 (2.2–5.6)	4.7 (2.9–6.6)	χ ^2^ = 0.388	0.53
Mainland	96.1 (94.4–97.8)	95.3 (93.4–97.1)		
**Mean age + SD (months)**	47.6 (46.5–48.6)	46.7 (45.6–47.9)	*t =* 1.070	0.29

### Sample size

The study compared the efficacy of two different PZQ treatment strategies; single and spaced double dose. It was assumed that there would be a significant difference in the number of children cured between the two treatments after 1 month. It was expected that 1 month after treatment parasite infection would be reduced to 30% and 20% with the single dose and double dose, respectively [35, 36]. Using the formula of comparison of two proportions [37] with 90% power and considering a 10% difference between the two proportions to be at the 95% significance level, the total sample size calculated is n = 784 children. Each intervention group will therefore be n = 392 children. In order to account for a 20% loss to follow-up; the sample was increased to 490 children in each treatment arm.

### Ethical statement

Ethical approval and clearance were obtained from Makerere University, Ethics Committee and the Uganda National Council for Science and Technology (Ref. No. UNCST-HS1274), respectively. The trial was registered with ClinicalTrial.gov, identifier: NCT01901484. The aim and procedure of the study were explained to the parents/guardians of the recruited children in the local language (Lusoga), and consent obtained before PZQ chemotherapy. Participation was voluntary and the parents/guardians had the right to withdraw their child/children at any time point from the study. Treatment was administered by trained medical personnel blinded to the children’s intervention group in the presence of each child’s caretaker. Children were closely monitored by trained and authorized staff for 30 minutes after drug administration and the caretakers were asked to report unusual behavior of their children within 24 hours. In case of vomiting and diarrhea, sachets of oral rehydration salt were given to the caretakers together with advice on how to dissolve the salt and administer the solution to the children.

### Parasitological examination

Participant children were asked to provide one stool sample on three consecutive days. Multiple stool collections were proposed due to day-to-day variation in egg counts of *S*. *mansoni* [38, 39]. Two slides of 41.7mg Kato-Katz [40] thick smears were prepared from each stool specimen and examined under a microscope (10x) to determine *S*. *mansoni* egg counts. For quality control, 10% of the prepared slides were randomly selected and re-examined by an independent expert microscopist. In case of discrepancies, the slides were reread and consensus obtained. Three stool specimens were collected again from each child both at a follow-up of 1-month and 8 months after the second treatment to determine PZQ efficacy and re-infection rates, respectively. Similar parasitological procedures used at baseline were applied in the follow-up surveys.

### Praziquantel intervention

Following parasitological screening, egg positive children were randomly assigned to two treatment groups; one receiving a single and the other a double dose. Children were weighed using a portable digital scale (Seca 771, Seca GmbH, Hamburg, Germany; accuracy: 0.1 kg) and offered a standard oral dose of PZQ (40 mg/kg body weight). The brand used was PZQ CIPLA 600 mg (Kundaim, India). The required dose can be administered relatively accurately because each PZQ tablet (600mg) is scored with 3 lines and can thus be subdivided into 4 equal parts of 150 mg [41]. The tablets were crushed [42] and mixed with drinking water to facilitate oral uptake in small children [15]. Before drug administration, children were given a piece of bread, and orange juice was provided after drug administration to minimize gastrointestinal side effects [43–45] and mask the bitter taste of PZQ [19], respectively. Two weeks after the first treatment, children belonging to the double dose group were given a second dose of PZQ (40 mg/kg of body weight). In case of vomiting, the children were offered a second dose. Treated children remained under supervision for a period of 30 minutes to monitor any immediate reactions as a result of drug administration and the caregivers were further encouraged to report any treatment-related symptoms observed in the children 24 hours post-treatment. In case of adverse events, the affected children would be referred to the nearest local health services.

### Statistical analysis

Data were double entered and cross-checked using EpiData version 3.1 (The EpiData Association, Odense, Denmark) and Microsoft Excel spread sheet software. Statistical analysis was carried out in Stata/IC release 12.0 (StataCorp; College Station, TX, USA). Intensity of infection (expressed in eggs per gram of stool, EPG) was calculated by multiplying the mean for the six slides by a factor of 24. Infection intensities were classified according to WHO guidelines [8]; as light: 1-99EPG, moderate:100-399EPG and heavy: ≥400EPG. The effects of treatment on geometric mean intensity (GMI) of infection were assessed by determining the change in GMI egg counts among all the children at 1 month post treatment, irrespective of whether they remained egg positive or became egg free. At-test for log transformed EPG at baseline and 1 month post treatment was used. Test of proportions of interest were calculated and comparisons made using the Pearson chi-square (χ^2^) test. The parasitological CRs for the two treatment regimens were calculated as the proportion of children with no egg excretion after treatment among those with eggs in their stool at baseline. CRs obtained with the two treatment regimens were compared using χ^2^. ERRs were determined by comparing the GMI egg output at baseline and 1 month after the second treatment (1–[GMI after treatment/GMI before treatment]) x 100). The proportions and 95% confidence intervals (95% CI) of children who were egg-negative 1 month post treatment but became positive at the 8 month follow-up were generated to compare re-infection between children who had received single and double dose treatments. Univariate and multivariate logistic regression analyses were used to assess relationships of sex, age group, intensity of infection and treatment dose with CR of *S*. *mansoni* infections using χ^2^ test. Adjusted odd ratios at 95% confidence interval were used [46]. P-values <0.05 were considered to indicate statistical significance.

## Results

### Baseline enrolment

During the baseline parasitological survey, 1,203 children were found infected with *S*. *mansoni*, but only 1,017 were randomized to treatment due to lack of informed consent or less than three stool specimens in 186 children (Fig 2). Before treatment another 292 were lost resulting in 725 receiving treatment (375 and 350 in the single and double dose group, respectively). The children that remained for PZQ treatment were comparable to the children lost to follow-up with respect to age, sex, and *S*. *mansoni* intensity of infection (Table 2). At the 1 month follow-up, 696 children were investigated for infection, 357 from the single dose group and 339 from the double dose group. The final cohort at 8 months follow-up consisted of 463 children; 236 and 227 children in the single and double dose group, respectively (Fig 2). A total of 554 children did not complete the study due to refusal of caregivers, absenteeism on the treatment/survey day or inaccessibility of habitation during the rainy season.

**Table 2:** Comparison of preschool-age children (n = 1017), who after randomization, were lost to follow-up or were retained in the study with regard to age, sex and infection intensity.

Characteristic	Lost follow-up n = 292 (%)	Retained in study n = 725 (%)	P-value
**Sex**			
Girl	132 (45.2)	358 (49.4)	0.23
Boy	160 (54.8)	367 (50.6)	
**Age group (months)**			
12–24	35 (12.0)	58 (8.0)	0.19
25–36	62 (21.2)	151 (20.8)	
37–48	73 (25.0)	209 (28.8)	
49–60	122 (41.8)	307 (42.3)	
**Infectionintensity (epg)**			
Light (1–99)	168 (57.5)	422 (58.2)	0.47
Moderate (100–399)	75 (25.7)	164 (22.6)	
Heavy (≥400)	49 (16.8)	139 (19.2)	

### Parasitological cure rate

Table 3 summarizes CRs of children who received a single and double dose of PZQ (40 mg/kg). At 1 month post-treatment follow-up, 84.3% children (587/696) were tested egg negative for *S*. *mansoni* and therefore considered cured. The results show that more children were cured with a double dose 85.5% (290/339) compared to 83.2% (297/357) cured by a single dose although this difference was not statistically significant (P = 0.39). There was a significant difference (P = 0.01) in CRs between double (81.8%) and single dose (62.0%) interventions of the moderately infected children. Children in the age group 12–24 months were all cured by either a single dose (25/25) or double dose (33/33), while the lowest CRs (single dose: 75.8%; double dose: 80.0%) were observed in the oldest age group of 49–60 months. Table 4 shows the results of the univariate and multivariate regression analyses on the relationships of CR with sex, age group, intensity of infection and treatment dose. Children had equal chances of being cured by either one or two doses of praziquantel (OR: 1.6; 95% 0.9–2.5; P = 0.07). There was no difference in CRs between girls and boys in any of the treatment arms. Older children, 49–60 months old, were more likely to remain infected after treatment with praziquantel (OR: 0.3; 95% CI 0.2–0.6; P <0.01) than their younger counter parts. Children with moderate and heavy intensity infections at baseline were more difficult to cure from infection (OR: 0.1; 95% CI 0.1–0.2; P<0.01) than those with light-intensity infection.

**Table 3:** Comparison of cure rates of praziquantel between single and double doses assessed 1-month post-treatment with regard to sex, age and *S*. *mansoni* infection intensity.

	Single dose (n = 357)		Double dose (n = 339)			
Characteristic	n	No. Cured (%)	n	No. Cured (%)	χ ^2^	*p*-value
**Sex**						
Girl	181	150 (82.9)	163	134 (82.2)	0.03	0.87
Boy	176	147 (83.5)	176	156 (88.6)	1.92	0.17
**Age group (months)**						
12–24	25	25 (100)	33	33 (100)		
25–36	67	57 (85.1)	79	69 (87.3)	0.02	0.90
37–48	116	100 (86.2)	87	76 (87.4)	0.06	0.81
49–60	149	113 (75.8)	140	112 (80.0)	0.73	0.40
**Pre-treatment intensity (epg)**						
Light (1–99)	225	220 (97.8)	191	182 (95.3)	1.97	0.16
Moderate (100–399)	79	49 (62.0)	77	63 (81.8)	7.54	0.01
Heavy (≥ 400)	53	28 (52.8)	71	45 (63.4)	1.40	0.24
**Overall**	**357**	**297 (83.2)**	**339**	**290 (85.5)**	**0.73**	**0.39**

**Table 4:** Regression analysis of factors associated with cure rate among preschool-age children.

Variable	Univariate COR (95% CI)	*p*-value	Multivariate AOR (95% CI)	*p*-value
**Treatment Arm (ref: single dose)**				
Double dose	1.2 (0.8–1.8)	0.39	1.6 (0.9–2.5)	0.07
**Sex (ref: boy)**				
Girl	1.3 (0.9–1.9)	0.20	1.5 (0.9–2.4)	0.08
**Age of the child (months) (ref: 12–36)**				
37–48	0.3 (0.2–0.6)	0.01**	0.7 (0.4–1.4)	0.34
49–60	0.6 (0.3–1.2)	0.15	0.9 (0.5–1.9)	0.91
**Intensity-epg (ref: light (1–99)**				
Moderate (100–399)	0.1 (0.1–0.2)	0.01**	0.1 (0.1–0.2)	0.01**
Heavy (≥400)	0.1 (0.0–0.1)	0.01**	0.1 (0.0–0.1)	0.01**

NB: COR, Crude Odds ratio; AOD, Adjusted Odds ratio; CI, Confidence Interval;

** Significant at P<0.01;

### Reduction in egg intensity

Table 5 shows a comparison of ERRs between single and two doses assessed 1-month after PZQ intervention. The highest ERR of 99.3% (95% CI:99.2–99.5) was observed in the double dose treatment in which GMI of infection reduced from 105.2 EPG at baseline to 0.70 EPG at 1 month after chemotherapy. In contrast, ERR in the single dose group was 98.9 (95% CI: 98.7–99.1), with *S*. *mansoni* GMI reduced from 79.5 EPG at baseline to 0.86 EPG 1 month after treatment. Difference in rates of egg reduction between single and double dose treatment regimen was significant (P<0.01). Overall intensity of infection at baseline was significantly higher (P = 0.008) in children in the double dose treatment arm. There was no significant difference (P = 0.94) in intensity of infection among children who received either a single or two praziquantel doses 1 month post treatment.

**Table 5:** Comparison of egg reduction rate between single and double doses assessed 1-month post-treatment.

				Egg reduction rate (ERR)		
Treatment group	Sample size	GMI epg (baseline)	GMI epg (one month follow-up)	ERR (%)	95% CI	P value
Single dose	357	79.5	0.86	98.9	98.7 99.1	
Double dose	339	105.2	0.70	99.3	99.2 99.5	0.01

### *S*. *mansoni* re-infection 8 months post-treatment

**Fig 3:**
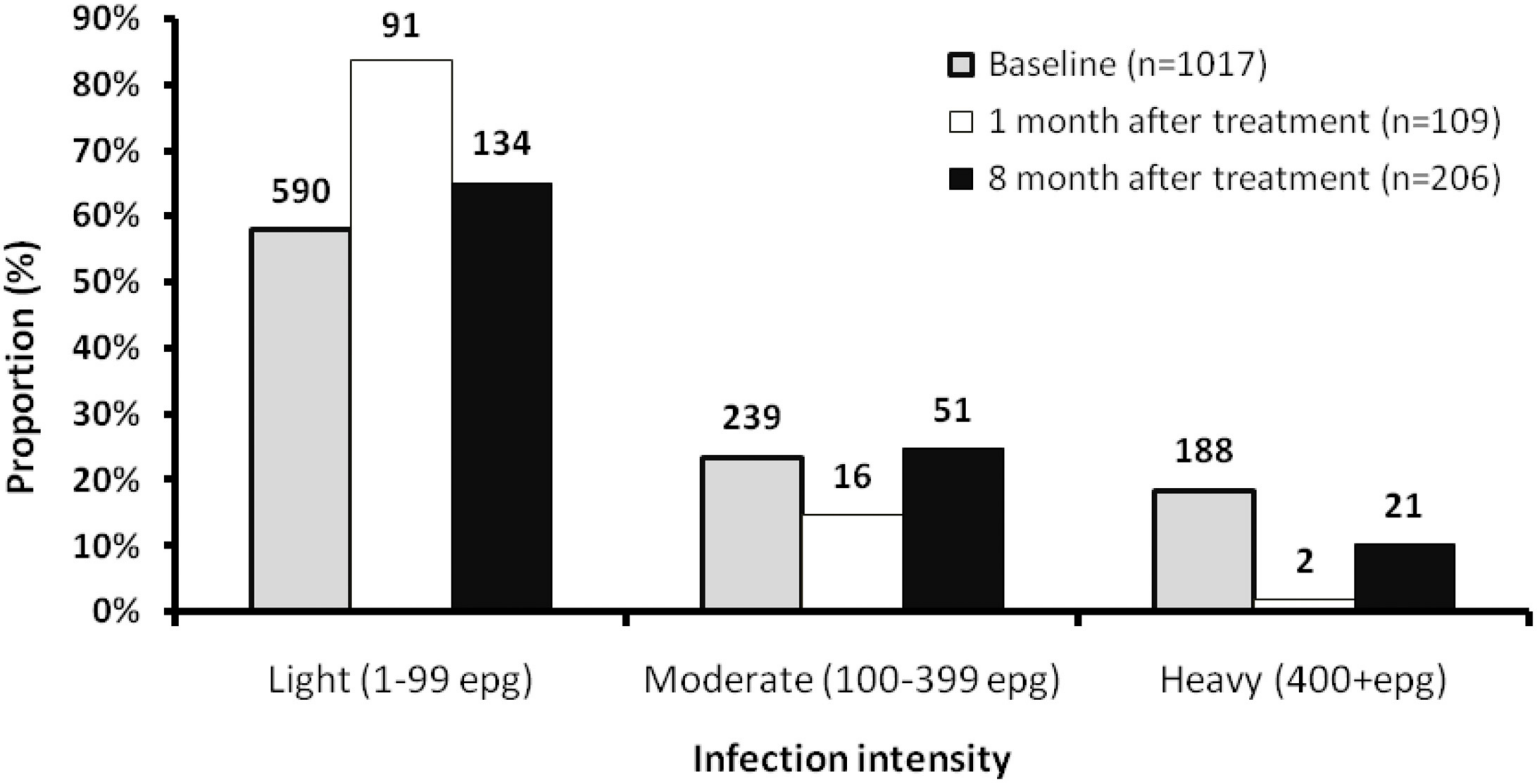
Overall distribution of *Schistosoma mansoni* infection intensity in preschool-age children at baseline, 1 month and 8 month after PZQ.

Table 6 shows the prevalence of re-infection in children. A total of 206 out of 463 (44.5%) children that were egg negative at 1-month follow-up were tested egg positive for *S*. *mansoni* 8 months later. There was no significant difference (P = 0.22) in re-infection rate comparing single dose children 92/236 (39.0%; 95% CI: 32.7–45.3) and double dose children 114/227 (50.2%; 95% CI: 43.7–56.8). Re-infection was non-significantly higher in boys than in girls. Re-infection intensity at 8 months post treatment was 4.0 EPG (95% CI: 2.9–5.8) and 7.1 EPG (95% CI: 5.1–9.8) for children who received a single and double dose, respectively; significantly higher (P = 0.03) among children who received a double dose treatment. Majority of infections at all 3 assessment points were of light intensity; at baseline (58%), 1-month (83%) and 8 months (65%) follow-ups (Fig 3). There was a marked reduction in heavy infection after treatment but it rose again to half its original level 8 months later (Fig 3).

**Table 6:** Comparison of *S*. *mansoni* prevalence of re-infection between single and double dose treatments, stratified by sex and age group,8 months post-treatment.

Characteristic	Single dose (n = 236)		Double dose (n = 227)	
	No. re-infected (%)	95% CI	No. re-infected (%)	95% CI
**Sex**				
Girl	44 (36.7)	27.9–45.4	49 (45.8)	36.2–55.4
Boy	48 (41.4)	32.3–50.5	65 (54.2)	45.1–63.2
**Age group (months)**				
12–24	5 (38.5)	7.9–69.1	12 (41.4)	22.3–60.4
25–36	18 (47.4)	30.7–64.0	24 (34.8)	23.3–46.3
37–48	30 (38.5)	27.4–49.5	28 (51.9)	38.1–65.6
49–60	39 (36.4)	27.2–45.7	50 (66.7)	55.7–77.6
**Overall**	**92 (39.0)**	**32.7–45.3**	**114 (50.2)**	**43.7–56.8**

### Side effects following PZQ treatment

Abdominal discomfort and diarrhoea were observed and reported within 30 minutes to 24 hours after drug administration. The symptoms were, however mild and short-lived, many subsided in less than three hours. Abdominal pain and diarrhoea were mostly reported in children aged 3–5 years. These side effects were mainly observed during the first PZQ administration with a single dose and not after administering the second dose. No severe adverse events were reported.

## Discussion

This study compared the efficacy and effect of one versus two spaced standard doses of PZQ (40 mg/kg body weight) in preschool-age children. Generally, the study revealed that PZQ in its current formulation is acceptable and efficacious in treating the young infected children. The overall CR (84.3%) and ERR (99.1%) obtained in this study are in the range of CRs/ERRs found in earlier trials [25, 47, 48] using 40 mg/kg PZQ dose. Although PZQ efficacy obtained in this study is satisfactory, it should be noted, however, that splitting or crushing tablets leads to dose inaccuracy due to reduced drug weight and poor absorption of the drug [49, 50]. The split and crushed tablets administered to the children in the present study could have lost their actual weight thus reduced dosage leading to therapeutic failure and hence final inaccurate CRs. This entails in not having proper PZQ formulations for children less than 6 years of age [15].

Treatment with PZQ leads to reduction in number of schistosome eggs via reduction in worm loads. This study presents significant ERRs in children who received two spaced standard doses of PZQ treatments (99.3% (95% CI: 99.2–99.5) compared to those that received a single treatment (98.9% (95% CI: 98.7–99.1). A similar trend was reported by other researchers comparing single and double dose PZQ efficacy in preschool-age children, school-age age children and adults [36, 43, 51, 52]. As mentioned earlier, PZQ is effective against mature schistosome worms but not juveniles [29, 53, 54]. It can be argued that the second treatment given two weeks later was able to target the now fully developed egg-laying adult worms which escaped drug action in the first treatment resulting into fewer eggs being laid and hence a higher ERR with the double regimen. This could be the case especially in the heavily infected children.

CR is also influenced by the infection worm load/intensity of *S*. *mansoni* infection prior to treatment [55, 56]. Indeed, in this study, there was a notable association between *S*. *mansoni* infection intensity at baseline and CR; children with light (1–99 EPG) infection intensity had the highest cure rate despite of whether they received a single or double dose. However, the importance of a second dose was appreciated as the intensity of infection increased due to the fact that children who were moderately (100–399 EPG) and heavily (400 and more EPG) infected had a higher CR with a double dose than with a single dose. Other studies [36, 51, 52, 56] have reported similar findings of increased CRs using two doses of PZQ. A CR difference between the two treatment regimens is also demonstrated in the varying age groups; children in age group 12–24 months were all cured with either single or double dose, while the rest of the age groups 25–36, 37–48 and 49–60 months who usually have higher infection intensities tend to have a higher CR with a double dose (CR: 87.3%, 85.4%, 75.2%) than with a single dose (81.9%, 83.3% and 71.5%), respectively. Older children aged 49–60 months are more exposed to schistosome infected water [10] and thus get more worm loads difficult to clear with a single PZQ intervention. They might require two doses. On the other hand, young children who are usually less exposed are lightly infected and therefore benefitted more from only a single treatment.

Total prevalence of re-infection 8 months post-treatment was 44.5%. It has been hypothesized that, after repeated rounds of infections and PZQ chemotherapy humans slowly acquire protective immunity to *S*. *mansoni* leading to partial resistance to re-infection [57, 58]. Treatment with PZQ boosts adult worm immunoglobulin E (IgE) antibodies which are associated with resistance to re-infection [59, 60]. Based on this hypothesis children aged 1–5 should experience high re-infection because they have not been exposed to *S*. *mansoni* long enough to produce schistosome resistant antigens as acquired immunity against schistosomes develops slowly over several years [61–63]. We had expected though that children receiving double treatments would experience a lower re-infection rate and/or intensity than those receiving only a single treatment because double treatments could delay the susceptibility to re-infection. However, this was not the case and was similar to other studies in Uganda performed in school-age children and adults [36]. On the other hand baseline infection intensity has been significantly associated with *S*. *mansoni* re-infections [64]. It is not surprising, therefore, that children in the double treatment arm who had heavy infection distribution at baseline turned out to be more re-infected 8 months post treatment. It has also been pointed out that increased resistance to re-infection is induced by repeated rounds of PZQ treatment and this was not the case with children in this study who were being treated for the first time [57].

Children in the double dose regime experienced more (50.2%) re-infection than those who received a single dose (39.0%) although this difference was not significant. However, the intensity of infection 8 months post-treatment was significantly higher (P = 0.03) in children who received two doses of PZQ. This means that at 8 months post treatment the double dose did not offer any further protection against schistosomes. It is also noted that incidentally at baseline, children in the double dose treatment arm were significantly more intensely infected that those in the single dose arm. Probably, double dose children had more residue immature worms that survived both treatments and these fully matured in the 8 months period thus laying more eggs. New re-infections as well as old ones in the double dose arm could therefore have led to the increased intensity compared to the children in the single treatment arm who originally had lighter infections.

Mild and short lived side effects due to PZQ administration that are reported in this study may be attributed to the low *S*. *mansoni* infection intensities in the children aged 1–5 years. Previous studies show that the frequency and severity of side effects is proportional to the intensity of infection [65, 66] and that bloody diarrhoea commonly correlates to pre-treatment egg load [67]. The bloody diarrhoea is due to the dying schistosomes which are attacked within 15 minutes after oral intake of PZQ; the PZQ penetrates the worm skin and rapidly moves through tissues causing muscle contraction and bleeding [25, 27, 68]. The vomiting was probably mainly caused by the children’s unfavourable reaction to the bitter taste of PZQ [69, 70] which irritates the mouth and throat [69]. Many children will tend to reject the drug upon administration and end up gagging because it is very difficult to mask the unpleasant taste of crushed tablets. Another explanation for the few registered side effects observed in this study is the food (piece of bread and soft drink) that was given to the children before and after administering the PZQ, respectively. Other studies have also associated pre-treatment snack with reduced side effects of PZQ [11, 43–45]. However, more side effects were observed at the first treatment (because of the initial heavier worm burden) than two weeks later (low intensity) when a second dose was administered.

There was a huge loss to follow-up mainly due to absenteeism and migration. However, the number of children that were lost in both treatment groups were comparable; 273 children lost in the single dose and 281 children lost in the double dose group. A second dose of PZQ administered to children below 6 years does not add value to the CR or reduce the rate of re-infection with schistosomiasis. However, since these children live in endemic communities where transmission is permanent and the rate of schistosome infections is high, a second dose of PZQ could be useful as it leads to significant higher ERRs compared to a single dose indicating a reduction in worm burden and consequently reduction in subsequent morbidity [71, 72]. It is evident that preschool-age children can safely and effectively be cured by a standard oral dose of PZQ without serious side effects. This age group is, therefore, recommended for inclusion in the on-going Uganda schistosomiasis control programme. However, this would only be possible if a paediatric formulation of PZQ is in place as crushing of tablets and providing juice to mask the bitter taste will not be logistically possible in the current set up of the programme.

## Supporting Information

S1 Checklist Consort checklist. (DOCX)

S1 Text Uganda National Council for Science and Technology approval letter. (PDF)

S2 Text Research Ethics Committe approval, Makerere University; page 1. (PDF)

S3 Text Research Ethics Committe approval, Makerere University; page 2. (PDF)
