# Supplementary material for: Single Versus Double Dose Praziquantel Comparison on Efficacy and Schistosoma mansoni Re-Infection in Preschool-Age Children in Uganda: A Randomized Controlled Trial
Source: PLoS Negl Trop Dis. 2015 May 26;9(5):e0003796. doi: 10.1371/journal.pntd.0003796 (PMC4444284; doi:10.1371/journal.pntd.0003796)
Supplement: S3 Text — (PDF) [file pntd.0003796.s004.pdf]

Please summarize the proposed change and the rationale for it in a letter to the School of Medicine Research and Ethics Committee. In addition, submit three (3) copies of an updated version of your original protocol application- one showing all proposed changes in bold or 'track changes,' and the other without bold or track changes.

Reporting
Other events which must be reported promptly in writing to the School of Medicine Research and Ethics Committee include:
Suspension or termination of the protocol by you or the grantor
Unexpected problems involving risk to participants or others

Adverse events, including unanticipated or anticipated but severe physical harm to participants.

Do not hesitate to contact us if you have any questions. Thank you for your cooperation and commitment to the protection of human subjects in research.

Final approval is to be granted by Uganda National Council for Science and Technology.

Documents approved for use along with protocol:
- English and translated Informed Consent forms
- English and translated Questionnaires

MAKERERE UNIVERSITY
SCHOOL OF MEDICINE
APPROVED
VALID UNTIL
04 OCT 2013
RESEARCH & ETHICS COMMITTEE
P.O.BOX 7072, KAMPALA

Yours sincerely,

Prof. James Tumwine
Chairperson School of Medicine Research and Ethics Committee
