# Supplementary material for: Single Versus Double Dose Praziquantel Comparison on Efficacy and Schistosoma mansoni Re-Infection in Preschool-Age Children in Uganda: A Randomized Controlled Trial
Source: PLoS Negl Trop Dis. 2015 May 26;9(5):e0003796. doi: 10.1371/journal.pntd.0003796 (PMC4444284; doi:10.1371/journal.pntd.0003796)
Supplement: S1 Text — (PDF) [file pntd.0003796.s002.pdf]

Uganda National Council for Science and Technology
(Established by Act of Parliament of the Republic of Uganda)
UNCST

Our Ref: HS 1274 **19th November 2012**

Dr. Allen Nalugwa
Child Health and Development Centre
Makerere University College of Health Sciences
Kampala

Dear Dr. Nalugwa,

RE: RESEARCH PROJECT, "INTESTINAL SCHISTOSOMIASIS IN CHILDREN AGED 1-5 YEARS, MORBIDITY ASSESSMENT AND THE EFFECT OF PRAZIQUANTEL ON MORBIDITY-JINJA DISTRICT, UGANDA"

This is to inform you that the Uganda National Council for Science and Technology (UNCST) approved the above research proposal on **30th October 2012.** The approval will expire on **30th October 2013.** If it is necessary to continue with the research beyond the expiry date, a request for continuation should be made in writing to the Executive Secretary, UNCST.

Any problems of a serious nature related to the execution of your research project should be brought to the attention of the UNCST, and any changes to the research protocol should not be implemented without UNCST's approval except when necessary to eliminate apparent immediate hazards to the research participant(s).

This letter also serves as proof of UNCST approval and as a reminder for you to submit to UNCST timely progress reports and a final report on completion of the research project.

Yours sincerely,

Jane Nabbuto
for: Executive Secretary
UGANDA NATIONAL COUNCIL FOR SCIENCE AND TECHNOLOGY

LOCATION/CORRESPONDENCE

Plot 6 Kimera Road, Ntinda
P. O. Box 6884
KAMPALA, UGANDA

COMMUNICATION

TEL: (256) 414 705500, (256) 312 314800
FAX: (256) 414-234579
EMAIL: info@uncst.go.ug
WEBSITE: http://www.uncst.go.ug
