# Supplementary material for: Single Versus Double Dose Praziquantel Comparison on Efficacy and Schistosoma mansoni Re-Infection in Preschool-Age Children in Uganda: A Randomized Controlled Trial
Source: PLoS Negl Trop Dis. 2015 May 26;9(5):e0003796. doi: 10.1371/journal.pntd.0003796 (PMC4444284; doi:10.1371/journal.pntd.0003796)
Supplement: S2 Text — (PDF) [file pntd.0003796.s003.pdf]

MAKERERE UNIVERSITY

P.O. Box 7072 Kampala, Uganda
E-mail: rresearch9@gmail.com

Phone: 256 414 533541
Fax: 256 414 541036/0414 532204

COLLEGE OF HEALTH SCIENCES
SCHOOL OF MEDICINE

RESEARCH ETHICS COMMITTEE

October 5, 2012

Dr. Allen Nalugwa
Child Health and Development Centre

Category of review

[X] Initial review
[ ] Continuing review
[ ] Amendment
[ ] Termination of study
[ ] SAEs

Dear Dr. Nalugwa,

Re: Approval of proposal #REC REF 2012-162
"Intestinal schistosomiasis in children aged 1-5 years, morbidity assessment and the effect of praziquantel on morbidity-Jinja district, Uganda"

Thank you for submitting an application for approval of the above – referenced proposal
The committee reviewed it and granted approval for one year, effective October 05th, 2012. Approval will expire on October 4th 2013.

Continuing Review
In order to continue work on this study (including data analysis) beyond the expiration date, the School of Medicine Research and Ethics Committee must reapprove the protocol after conducting a substantive, meaningful, continuing review.
This means that you must submit a continuing report form as a request for continuing review. To best avoid a lapse, you should submit the request six (6) to eight (8) weeks before the lapse date. Please use the forms supplied by our office.

Amendments
During the approval period, if you propose any change to the protocol such as its funding source, recruiting materials, or consent documents, you must seek School of Medicine Research and Ethics Committee approval before implementing it.
